# Prenatal care and childbirth assistance in Amazonian women before and after the Pacific Highway Construction (2003–2011): a cross-sectional study

**DOI:** 10.1186/s12905-016-0316-4

**Published:** 2016-07-13

**Authors:** Andréia S. Guimarães, Saulo A. S. Mantovani, Humberto Oliart-Guzmán, Antonio C. Martins, José Alcântara Filgueira-Júnior, Ana Paula Santos, Athos Muniz Braña, Fernando Luís Cunha Castelo Branco, Thasciany Moraes Pereira, Breno Matos Delfino, Alanderson A. Ramalho, Cristieli S. M. Oliveira, Thiago S. Araújo, Carlos Hermogenes Manrique de Lara Estrada, Nancy Arróspide, Pascoal T. Muniz, Cláudia T. Codeço, Mônica da Silva-Nunes

**Affiliations:** Centro de Ciências da Saúde e do Desporto, Universidade Federal do Acre, Campus Universitário, BR 364, Km 04, Bairro Distrito Industrial, Rio Branco, AC Brazil; Dirección Regional de Salud de Madre de Dios, Av. Ernesto Rivero N° 475, Puerto Maldonado, Peru; Instituto Nacional de Salud, Cápac Yupanqui, Jesus María, Lima 11 1400 Peru; Programa de Computação Cientifica, Fundação Osvaldo Cruz, Avenida Brasil, 4365, Manguinhos, Rio de Janeiro, RJ Brazil

**Keywords:** Amazon, Prenatal care, Childbirth

## Abstract

**Background:**

Attention to prenatal care and child delivery is important for the health of women and children, but in the Amazon these indicators tend to be historically unfavorable, in part by geographical and political isolation. In 2003 both Brazilian and Peru governments have finished paving an international road connecting remotes areas in the Brazilian Amazon to the Pacific coast in Peru.

**Methods:**

The situation of prenatal care and child delivery with mothers of children under 5 years old living in the urban area of Assis Brasil, Acre was assessed in two cross-sectional studies performed in 2003 and 2011, corresponding to the period before and after the Pacific highway construction.

**Results:**

In 2003, most mothers were of black/Afro-American ethnicity, or “pardos” (the offspring of a Caucasian with a African descendant) (77.69 %), had more than 4 years of schooling (73.40 %) and had a mean age of 22.18 years. In 2011, the number of as a migration of indigenous women increased from 0 to 14.40 % of the respondents, because of migration from communities along the rivers to urban areas, with no other significant changes in maternal characteristics. No significant improvement in childbirth assistance was noticed between 1997 and 2011; only the percentage of in-hospital vaginal deliveries performed by doctors increased from 17.89 to 66.26 % (*p* <0.001) during this period. Access to prenatal care was associated with white ethnicity in 2003, and higher socioeconomic level and white ethnicity in 2011, while the higher number of prenatal visits was associated with higher maternal education and higher socioeconomic levels in 2011. Vaginal child delivery at a hospital facility was associated with maternal age in 2003, and year of birth, being of white ethnicity and higher level of education in 2011.

**Conclusions:**

The indicators of prenatal care and child delivery were below the national average, showing that geographical isolation still affects women’s health care in the Amazon, despite the construction of the highway and governmental health protocols adopted during this period.

## Background

Prenatal care is one of the most important issues in women’s health. Access to prenatal care needs to be done as early as possible, because it is during this time that maternal disease can be controlled and preventive actions can be performed on behalf of offspring [1]. Besides prenatal care, attention to childbirth is another very important issue, because more than 50 % of neonatal and maternal deaths occur during childbirth [2].

Institutional maternal health care in Brazil started in the 1940’s, and in 1994, the creation of the Family Health program allowed new guidelines to be put in place. Prenatal care coverage and institutional childbirth increased to 98.09 and 96.58 %, respectively. Other changes took place in 2006, with the governmental plan “Pact for Life,” in which basic health policies were reinforced. These policies included strengthening the public health system and decentralizing health services, aiming to reduce maternal health concerns [3].

Since then, the necessary conditions for the magnification of health posts and family health units have been created, resulting in better maternal health status. In Brazil, the attendance of at least one consultation during pregnancy has reached 98.09 % [4]. However, this improvement was not homogeneously achieved in the whole country because of regional inequities.

The Amazon region is an area with the highest fertility rate in Brazil and the third highest rate of child mortality. In 2009, there were 29 deaths per 1000 births in the Amazon against 19 deaths per 1000 births in the most developed region [5]. The Amazon also has the lowest wealth index, lowest human development index (HDI) and lowest educational levels [5]. Until 2000, the Amazon had the lowest rates of health posts per inhabitants in the country and one of the smaller rates of hospital beds per 10,000 inhabitants in the country [4].

Some cities in the North region (where the Amazon is located) are considered to have the lowest HDI in the country. Of 5565 Brazilian municipalities, 446 are located in the North region and 310 in the Amazon. About 54.2 % of them are in the lowest IDH quartile in the country [5], reflecting the social inequities of the Amazon region.

These particularities have created several obstacles for governmental health care assistance in the Amazon region. Although the Family Health program and other pacts for health were implemented in 2006, prenatal care coverage was only 89.74 % in the state of Acre in 2009 [4]. Childbirth assistance was also low, with only 85.70 % of the births occurring in hospital facilities [4]. It is possible that these numbers indicate the difficulties in accessing health care in the Amazon, specially for rural and riparian communities.

In the state of Acre, there are still several municipalities that have no road access and can be reached only by air or water. To reverse this geographic isolation, several roads have been built in the Amazon region, and in 2001, the Interoceanic Road began to be paved, linking Brazil, Peru and Bolivia, with an estimated cost of US$ 810 million [6]. This road was the second one to be paved in the state of Acre. Between December 2002 and March 2003, a few cities started to have permanent road access for the first time ever. Assis Brasil was one of this cities [7]. Until 2003, Assis Brasil’s inhabitants had depended on a non-paved road to reach neighboring cities and the capital, Rio Branco, in a several-day journey. At that time, high risk child delivery and pregnant women in need of a cesarean surgery were referred to the neighboring city which was assessed by 100 km of unpaved road, making access to optimal health care services difficult, and sometimes impossible, such as in the rainy season [8]. The aim of this study was to assess changes in prenatal care and childbirth assistance in the municipality of Assis Brasil, Acre, before and after the Pacific road was constructed, assuming that the road might have improved health care access to women

## Methods

### Study area and population

#### Study area

Assis Brasil was founded in 1976 from old established communities in the areas of rubber plantations. Located in the Acre River Valley and 344 miles southwest of Rio Branco, Assis Brasil occupies an area of 4974 km^2^. It borders the municipality of Brasileia to the east, the cities of Iñapari (Peru) and Bolpebra (Bolivia) to the south and the municipality of Sena Madureira to the north. The climate is equatorial hot and humid, a subdivision of the tropical climate [9]. A map of the area has been published elsewhere [10]. In 2003, the population was estimated at 3667 inhabitants, and 38 % lived in rural areas. In 2010, Assis Brasil had 6072 inhabitants (3091 males and 2981 females), and 10.24% were between 0 and 4 years old [11]. The percentage of the population that was living in urban areas was estimated at 61% in 2010 [11]. The HDI of Assis Brasil was 0.588 in 2010, being in the lowest quartile among all Brazilian cities [5].

#### Study design, population studied and outcomes

Since the objective of the study was to evaluate changes in the municipality of Assis Brasil over the decade fostered by the paving of the road, the study was performed at two times: 2003 and 2011. This was done to cover for the period before and after the paving. By using health care worker registries of all children under 5-years old living in the urban area in January 2003 and January 2011, we identified pregnancies and deliveries (and their respective mothers) that occurred from 1997–2003 and 2006–2011. Due to logistic limitations, we were not able to assess prenatal care and delivery registries, since informatized record keeping in the Amazon is very recent and older, manual records are very scarce. Therefore pregnancies that resulted in abortions, stillbirths or neonatal deaths were missed. Also, inclusion criteria entailed being able to interview the biological mothers. We were not able to interview mothers that moved from the urban area of Assis Brasil to rural or riverine areas or to other municipalities between 1999 and 2011.

In 2003, we identified 200 children between 0 and 5 years old living in the city, of which 174 (87 %) were still being raised or in contact with their biological mother. From those, we were able to interview 154 biological mothers (77.0 %). In 2011, we identified 454 children between 0 and 5 years old that were living in the urban area, but only 412 (90.74 %) were being raised by their biological mothers. We were able to interview all 412 mothers.

Data collection occurred using questionnaires to investigate the following variables: socioeconomic and demographic characteristics (wealth index, receipt of benefits, living with a partner, ethnicity, education and religion); maternal reproductive history (age at first pregnancy, age at current pregnancy, number of pregnancies/childbirth, number of stillbirths and number of neonatal deaths); maternal morbidity and behavior (smoking and consumption of alcoholic beverages during pregnancy, gestational morbidities) and year of pregnancy and childbirth. For pregnancies that started in 1 year and ended in the following year, the year of pregnancy was considered the one in which most of the pregnancy occurred. Information on education, religion and ethnicity was gathered as that of current status, since the possibility of changes in those variables was minimal during the study. All questionnaires were administered by the research team.

The outcomes assessed were attending prenatal care (yes or no), number of prenatal consultations (equal to/more than six consultations or less than that) and vaginal delivery of a child inside a hospital facility (yes or no). The number of six consultations was adopted because Brazilian Ministry of Health (MoH) protocols used at the time of the pregnancies defined “optimal care” as having attended least six consultations [3], although this definition is not broadly used in other countries, Also, one of the MoH’s requisites for optimal child delivery at the time of the study was having a delivery with a trained health professional inside a hospital facility [3].

### Statistical analysis

#### Wealth index construction

To assess the family socio-economic status, a household wealth index was created, based on the presence of twenty-one consumer goods and household appliances (television, stereo, DVD player, gas stove, refrigerator, washing machine, telephone, bicycle, blender, electric iron, car, sofa, satellite dish, mobile phone, motorcycle, computer, boat, motor boat, water well, power generator and microwave oven) as described in previous publications/studies [9, 12].

Principal component analysis (PCA), carried out using XLSTAT software, version 7.5.2 (Addinsoft, New York, NY), with the parameters covariance (n-1) and correlation biplot/coefficient = n, was used to weigh each variable, as described by Filmer and Pritchett (2001) [13]. The variables were standardized, and PCA analysis was performed for each year. To exclude variables that did not have a major contribution in the analysis, we used the Jolliffe method (1972) [14], adapted for the covariance matrix, thus excluding variables with variance lower than 0.7. For 2003, only five variables remained in the analysis, and for 2011, twelve variables were maintained in the score. For 2003, the first principal component explained 61.11% of total variance. The highest scores were given for the possession of satellite dish (0.814), sofa (0.610) and blender (0.507). The lowest scores were given to families without a refrigerator (−0.983) and washing machine (−0.740). For 2011, the first principal component explained 37.87% of total variance, and the highest scores were related to the ownership of a computer (0.824), a car (0.753) and a stereo (0.320). The lowest scores were given to families without television (−0.916) and refrigerator (−0.838). The scores for each variable were added to estimate the household wealth index. The range was−3.552 to 2.761 in 2003 and−5.201 to 4.104 in 2011, and these indices were used for stratification into quartiles (first quartile, 25% richer) and recategorization into two groups (the poorer half and the richer half). For each year, this procedure was performed for all children versus only one child per household, in order to check for non-independency of the individual information. There was no statistical difference in the comparison of the indices calculated by each technique; we thus opted to use the index per household instead of the index per child. The data were entered into SPSS 13.0 software (SPSS Inc., Chicago, IL) and transferred to R 2.14 software (R Development Core Team, 2011) for statistical analysis. Student’s t-test was used to compare means and a chi-square test for comparing frequencies or proportions with α = 0.05 critical level.

Factors associated with the outcomes were identified in two stages. In the first stage, univariate logistic regression analysis was applied and independent variables that were associated with outcomes with *p* < 0.20 values were selected to compose the multiple models. In the second stage, the factors associated with the outcomes were identified through a hierarchical multiple logistic regression analysis, using a conceptual model and adapted procedures from previous studies [15]. The model included a distal block and a proximal block. The distal block included sociodemographic and maternal characteristics that were constant throughout the years (socioeconomic index, ethnicity, maternal education, maternal religion). The proximal block included maternal or family characteristics that varied between pregnancy events (age at current pregnancy, number of pregnancies, child delivery, abortions and stillbirths, living with a partner at the time of pregnancy, consumption of alcohol and tobacco during pregnancy). Year of pregnancy or childbirth was included in all final models as confounding factor, since access to health care may have changed throughout the years.

Analyses of the multiple regression models were carried out from the more distal to the more proximal block. All variables selected at the univariate stage were added to each block. The variables that showed *p* < 0.05 by the Wald test were selected as a variable associated with its outcome and remained in the multiple regression models in the analyses of subsequent blocks. The variables with p ≥ 0.05 were excluded and evaluated if they could reduce the model’s explanatory power. In the cases of reduction of explanatory power above 10 %, the variable was reinserted and kept in the multiple regression model. It was not possible to adjust the multiple regression models for 2003 since there was a small number of participants; for this year, we presented the results of the univariate analyses.

The goodness of fit was assessed by either analysis of variance, Akaike’s information criterion (AIC) values or odds ratio (OR) changes. Interaction terms were evaluated, and diagnostic tests (Cook’s distance for influential points and studentized residuals) were applied using the package car of the R 2.14.0 software. No statistically significant interaction terms were identified. No important influential points were identified for any of the variables included in the final model.

Since ethnicity was the only variable associated with having access to prenatal care and indigenous people is an important ethnic group in the Amazon, a separate analysis was performed considering only indigenous women and the outcome having access to prenatal care. When performing this separate analysis, it was possible to detect new variables associated with not having access to prenatal care that were not detected before among the general group of women. This analysis is presented separatedly.

#### Modeling multiple pregnancies in the same subject

For the outcomes “number of prenatal consultations” and “having a vaginal delivery inside hospital facility,” some of the women included in the 2011 study had more than one pregnancy/child delivery within the last 5 years, and therefore had attended more than one prenatal care appointment or had more than one child delivery within that period. In these situations, some of the socioeconomic and maternal variables included in the study were shared by different pregnancies/children, and models that assume independent observations could overestimate or underestimate the size of the associations.

We therefore adopted three strategies of analysis. The first strategy included only one pregnancy/childbirth per mother (the more recent one), where generalized logistic regression models with only fixed effects were used. This model resulted in only 238 vaginal deliveries included in the study for the outcome of normal childbirth given at a hospital. The model also resulted in 365 subjects included for the outcome of attending prenatal care, and it did not have enough power to detect significant associations.

The second strategy included all pregnancies/childbirths per mother. Mixed-effects logistic regression models with multivariate normal random effects were fitted using penalized quasi-likelihood in the R 2.14 ’s MASS library. Clustering into mothers/households was treated as a random effect in the model), while all other covariates were entered as fixed-effect variables. Only the variables associated with statistical significance at the 5% level and those that altered the parameter estimate of fixed-effects variables (odds ratio) or the unexplained residual effects at household level (random-effects estimation) by 10% or more were retained in these final models. With this approach, 362 pregnancies from 298 mothers and 324 vaginal deliveries from 254 mothers were included in the analysis.

There was a possibility that some degree of distortion in effects estimation could still occur since some of the maternal/household variables changed according to the pregnancy while others did not. We therefore adopted a third strategy where variables from distal and proximal blocks as described in the conceptual model were included in separate models. (mixed model 1 = wealth index, ethnicity, maternal schooling and maternal religion; fixed model 2 = age at time of pregnancy, number of pregnancies, child delivery, stillbirths and abortions, living with a partner during pregnancy and consumption of alcohol and tobacco during pregnancy) We then compared strategies 2 and 3. No significant differences were found in odds ratio values, confidence intervals and *p* values between strategies 2 and 3, so we chose to present the results of the second modeling strategy.

For the year 2003, only two mothers had more than one pregnancy/childbirth, so we performed generalized linear regression modeling using only univariate analysis due to reduced sample size (*n* = 154). Very few subjects declared themselves as of indigenous ethnicity, so all women, despite their ethnicity, were included in the same model.

## Results

### Health service characteristics in 2003 and 2011

In 2003, the Unified Health System in Assis Brasil was composed of three units (health center, an indigenous health care center and a mixed unit with inpatient and outpatient services) that provided medical services (one medical doctor), dentistry services (one dentist), nursing services (one nurse) and technical support for them, as seen in Table 1. Between 2003 and 2011, a basic health unit, based on the principles of family health, was created. Human resources were also improved, with the arrival of both college graduates and technicians. The basic healthwork teams at the basic units incorporated a new dentist, a new medical doctor, a physiotherapist, another nurse, two pharmacy clerks, four nursing technicians and eleven community health agents. The mixed-unit healthwork team also changed, incorporating three new medical doctors, three nurses, one pharmacist, one biochemist and three nursing technicians. Therefore, both the basic health units and health center had a significant improvement in the qualifications and number of health care workers. Another significant improvement was the implementation of follow-up services for hypertension and diabetes and testing for inherited metabolic diseases. Prenatal care was provided by a medical doctor, whenever there was one hired by the service, or a registered nurse. Institutional child delivery was provided by medical doctors, midwives or nurses. Non-institutional child delivery was usually provided by a midwife or family member.

**Table 1:** Characterization of the health network of the city of Assis Brasil in 2003 and 2011

Variables	2003	2011
*Number of health units*		
Health Centre	01	01
Basic Health Unit	00	01
Indigenous Health Care Unit	01	01
Mixed Unit	01	01
*Number of professionals (Basic Health Unit)*		
Community Health Agent	06	17
Pharmacy Clerk	00	02
Dental technician	01	02
Nursing Assistant/Nursing Technician	02	06
Dentist	01	02
Nurse	01	02
Physiotherapist	00	01
Medical Doctor	01	02
*Number of professionals (Mixed Unit)*		
Pharmacy Clerk	01	01
Nursing Assistant/Nursing Technician	08	11
Biochemist	01	01
Nurse	01	04
Pharmacist	00	01
Medical Doctor	02	05
Laboratory Technician	02	02
X-ray Technician	02	02

### Characteristics of the study population in 2003 and 2011

Characteristics of biological mothers are shown in Table 2. In 2003, most of the women (77.69 %) were of “pardo” ethnicity (the offspring of Caucasian and Black people), had more than 4 years of education (73.40 %) and were adept at one religion system (90.98 %). The average age during pregnancy was 22.18 years (minimum of 13 years and maximum of 40 years). Alcohol and tobacco consumption during pregnancy was 12.30 and 16.89 %, respectively. In 2011, there were important changes in ethnicity, because several indigenous women (14.40 %) migrated to the urban area between 2003 and 2011, without any other significant change in maternal characteristics.

**Table 2:** Characteristics of biological mothers. Assis Brazil, Acre, 2003 and 2011

Variables	2003 (all ethnicities)		2011 (all ethnicities)		*p* value^b^
	N (154)^c^	%	N (412)^c^	%	
*Religion*					0023
not adept	11	9.01	71	17.30	
adept	111	90.98	349	82.70	
*Ethnicity*					<0.001
indigenous	0	0.00	59	14.40	
Black	7	5.79	17	4.10	
“parda”	94	77.69	274	66.70	
White	20	16.52	61	14.80	
*Education*					0.292
0 to 4 years	28	26.93	105	28.60	
> 4 years	76	73.08	262	71.40	
*Maternal marital situation during pregnancy*					-
Lack of partner	-	-	82	20.00	
Presence of partner	-	-	327	80.00	
*Cigarette smoking during pregnancy*					0.072
yes	25	16.89	46	11.17	
no	123	83.11	366	88.83	
*Alcohol consumption during pregnancy*					0.427
yes	18	12.30	41	10.00	
no	128	87.70	370	90.00	
*Current gestational status*					-
multiparous	-	-	281	68.40	
primiparous	-	-	130	31.60	
*Previous abortions or stillbirths*					-
yes	-	-	73	18.90	
no	-	-	314	81,14	
*Number of previous pregnancies*	-	-	412	1.96^a^	-
*Age at current pregnancy (in years)*	-	22.18^a^	411	23.45^a^	0.853

^a^Mean

^b^Pearson’s chi-square test

^c^The *n* may vary due to non-response (missing)

Prenatal care was not homogeneous during the study period. In the 2003 study, only 33 % of the pregnancies analyzed were reported to have happened between 1997 and 1999, and the remaining pregnancies occurred between 2000 and 2002. In 2011, pregnancies were homogeneously distributed from 2006–2011. This non-homogeneous distribution of pregnancies may be related to internal migration from rural to urban areas as the road was being constructed.

### Maternal health care in 2003 and 2011

Table 3 compares maternal health indicators in 2003 and 2011. The major change regarded the health professionals that delivered assistance during childbirth: medical assistance during childbirth changed from 17.89 to 66.26 % (*p* < 0.001). All other indicators did not change significantly.

**Table 3:** Indicators of maternal care. Assis Brazil, Acre, 2003 and 2011

Indicators	2003		2011		*p* value^a^
	N (154)^b^	(%)	N (412)^b^	(%)	
*Had access to atal care*					0.300
no	10	6.50	18	4.40	
yes	144	93.50	394	95.60	
*Number of prenatal consultations*					0.841
less than 6	49	39.20	139	38.19	
equal to or greater than 6	76	60.80	225	61.81	
*Type of childbirth*					0.138
normal	125	82.78	326	79.13	
cesarean	26	17.22	86	20.87	
*Place of vaginal delivery*					0.485
in hospital	20	16.00	45	13.80	
outside hospital	105	84.00	281	86.20	
*Professional conducting the normal childbirth*					< 0.001
Nurse/midwife/other	101	82.11	110	33.74	
Medical doctor	22	17.89	216	66.26	

^a^ Pearson’s chi-square test

^b^ The *n* may vary due to non-response (missing)

### Access to prenatal care among all women

The socioeconomic index was associated with attending prenatal care in 2003 at a *p* value of 0.050. Being in the richest half of the study group increased the odds of attending prenatal care by 486 % (Table 4). No significant association was found between prenatal care and any other variable studied.

**Table 4:** Prenatal care and childbirth care in the year of 2003, according to maternal characteristics, Assis Brasil, Acre

Variable	N^c^	%	Non-adjusted	(95 % CI)	*p* value^b^
*ATTENDING PRENATAL CARE (n = 154)*					
*Ethnicity*					
Black/“parda”	101	92.08	1	-	-
White	20	100.00	-	-	-
*Socioeconomic index*					
Poorer half	73	89.04	1	-	-
Richer half	81	97.53	4.86	(1.23–69.00)	0.050
*Education*					
0 to 4 years	25	88.00	1	-	-
> 4 years	69	97.10	4.57	(0.72–29.14)	0.108
*Cigarette smoking during pregnancy*					
yes	25	88.00	1	-	-
no	123	95.93	3.22	(0.72–14.45)	0.127
*Year of prenatal care*					
1997 to 1998	38	86.84	1	-	-
1999 to 2002	117	94.87	3.36	(0.92–12.33)	0.067
*Age at current pregnancy (in years)* ^a^	22.18^a^	93.39	0.99	(0.70–1.13)	0.919
*ATTENDING SIX OR MORE PRENATAL CONSULTATIONS (n = 125)*					
*Ethnicity*					
Black/“parda”	76	65.79	1	-	-
White	20	65.00	0.97	(0.34–2.72)	0.947
*Socioeconomic index*					
Poorer half	56	53.57	1	-	-
Richer half	69	66.67	1.73	(0.84–3.58)	0.137
*Education*					
0 to 4 years	18	33.33	1	-	-
> 4 years	61	68.85	4.42	(1.44–13.55)	0.009
*Cigarette smoking during pregnancy*					
yes	21	57.14	1	-	-
no	100	61.00	1.17	(0.45–3.04)	0.743
*Alcohol consumption during pregnancy*					
yes	16	75.00	1	-	-
no	103	57.28	0.45	(0.14–1.48)	0.187
*Year of prenatal care*					
1997 to 1998	22	68.18	1	-	-
1999 to 2002	103	59.22	0.68	(0.25–1.8)	0.436
*Age at current pregnancy (in years)* ^a^					
	21.80^a^	65.96	0.97	(0.9–1.05)	0.486
*VAGINAL DELIVERY AT A HOSPITAL FACILITY (n = 125)*					
*Ethnicity*					
Black/“parda”	85	83.53	1	-	-
White	14	85.71	1.18	(0.24–5.88)	0.837
*Socioeconomic index*					
Poorer half	61	80.33	1	-	-
Richer half	64	85.94	1.50	(0.58–3.85)	0.404
*Education*					
0 to 4 years	20	80.00	1	-	-
> 4 years	55	90.91	2.50	(0.60–10.45)	0.209
*Cigarette smoking during pregnancy*					
yes	24	79.17	1	-	-
no	99	83.84	1.37	(0.44–4.19)	0.586
*Alcohol consumption during pregnancy*					
yes	16	87.50	1	-	-
no	105	81.90	0.65	(0.14–3.09)	0.584
*Year of prenatal care*					
1997 to 1998	47	82.98	1	-	-
1999 to 2002	78	83.33	1.03	(0.39–2.69)	0.959
*Age at current pregnancy (in years)* ^a^	22.25^a^	81.82	0.90	(0.82–0.98)	0.018

^a^Average

^b^Wald Test

^c^The *n* may vary due to non-response (missing)

In 2011, the main association was found between ethnicity and attending prenatal care. While only one non-indigenous woman did not attend prenatal care, the lack of prenatal care among indigenous woman was high (24 %). Because of that, an analysis including only indigenous women was performed.

### Access to prenatal care among indigenous women

Among indigenous women (*n* = 59), the only variable associated with prenatal care was year of pregnancy. Pregnancies that occurred between 2008 and 2010 had higher odds of accessing prenatal care than those occurring from 2005–2007 [OR – 26.78, Confidence Interval (CI) 95 % 3.09–232.19, *p* = 0003]. Although education did not have a significant association with prenatal care, it was an important variable for better model fitting (OR = 7.68, CI 0.81–72.77, *p* = 0.076). (see Table 5)

**Table 5:** Factors associated with prenatal care, considering the indigenous biological mothers only. Assis Brazil, Acre, 2011

Variables	Crude OR	(IC 95 %)	Adjusted OR^a^	(IC 95 %)	*p* value^b^
*Maternal schooling*					
0 to 4 years	1	-	1	-	-
> 4 years	5.87	(0.70–49.38)	7.68	(0.81–72.77)	0.076
*Year of prenatal care*					
2005 to 2007	1	-	1	-	-
2008 to 2010	23.56	(2.82–197.00)	26.78	(3.09–232.19)	0.003

^a^ *Odds Ratio* (OR) adjusted by all variables in the table, *n* = 59

^b^ Wald test

### Quality of prenatal care

Table 4 shows factors associated with six or more prenatal consultations for the 2003 study. The only variable with significant association was maternal education; women with more than 4 years of formal education were more likely to have attended at least six prenatal consultations (OR = 4.42, *p* = 0.009).

In the 2011 study, ethnicity, education and socioeconomic index were associated with the number of prenatal consultations (see Table 6). Non-indigenous mothers (OR = 7.00, *p* < 0.001), those with more than 4 years of education (OR = 2.97, *p* = 0.002) and those in the richer half of the study population (OR = 2.27, *p* = 0.001) were at increased odds of having attended at least six prenatal consultations. In other words, indigenous mothers and those with unfavorable socioeconomic conditions were less likely to have optimal prenatal care.

**Table 6:** Prenatal care and childbirth care in the year of 2011, according to maternal characteristics, Assis Brasil, Acre

Variables	Crude OR	(IC 95%)	Adjusted OR	(IC 95 %)	*p* value ^b^
*ATTENDING SIX OR MORE PRENATAL CONSULTATIONS (n = 361)* ^c^					
*Ethnicity*					
Black/“parda”	1	-	1	-	-
White	6.50	(2.90–14.57)	3.94	(1.67–9.30)	0.002
*Education*					
0 to 4 years	1	-	1	-	-
> 4 years	3.39	(2.10–5.46)	2.10	(1.21–3.67)	0.009
*Socioeconomic index*					
Poorer half	1	-	1	-	-
Richer half	2.63	(1.72–4.04)	1.72	(11.07–0.94)	0.026
*Age at current pregnancy (in years)* ^a^	1.03^a^	(1.00–1.07)	1.09	(1.04–1.15)	< 0.001
*Number of previous pregnancies*	0.87	(0.77–0.98)	0.80	(0.68–0.94)	0.009
*VAGINAL DELIVERY AT A HOSPITAL FACILITY (n = 324)* ^d^					
*Ethnicity*					
Black/“parda”	1	-	1	-	-
White	3.62	(1.85–7.11)	2.61	(1.24–5.51)	0.012
*Education*					
0 to 4 years	1	-	1	-	-
> 4 years	2.76	(1.48–5.12)	1.98	(1.0–3.94)	0.005
*Year of childbirth*					
2005 to 2007	1	-	1	-	-
2008 to 2010	3.80	(2.02–7.14)	3.60	(1.88–6.89)	< 0.001

^a^Average

^b^Wald Test

^c^ *Odds Ratio* (OR) adjusted by ethnicity, education, socioeconomic index, maternal age and number of previous pregnancies

^d^ *Odds Ratio* (OR) adjusted by ethnicity, education and year of childbirth

### Childbirth assistance

Table 4 also shows factors associated with childbirth assisted by a health professional inside the hospital in the 2003 study. Older women were less likely to seek the hospital for institutional childbirth occurring between 1997 and 2003. No association was found with socioeconomic and behavioral characteristics.

In the 2011 study (Table 6), childbirth year, ethnicity and maternal education were associated with giving birth at a hospital facility. Non-indigenous women (OR = 3.39, *p* = 0.093) having more than 4 years of formal education (OR = 5.74, *p* = 0.001) and giving birth between 2008 and 2010 (OR = 6.80, *p* < 0.001) were at increased odds of having childbirth at a hospital facility with a trained health professional.

## Discussion

### Access to prenatal care

According to MoH [4], the prenatal care coverage in 2003 was 96.79 % for the whole country, 93.43 % in the North region and 85.77 % in the state of Acre. The prevalence found in Assis Brasil in 2003 (93.50 %) is similar to that obtained in the North region and higher than that recorded for Acre. However, it is still lower than the national average. In 2009, national records show prenatal coverage of 98.09 % in Brazil, 95.79 % in the North region and 89.74 % in Acre. Since the prenatal coverage in Assis Brasil was 95.60 % in 2011, it was still below national coverage and even lower than average coverage for the North region. This low coverage, when compared with the national and regional coverage, can be explained by obstacles posed by environmental and geographical characteristics of the Amazon, especially for people living in rural and riverine areas. It also creates difficulty in fixing human health resources in remote areas. Despite the Pact for Life [2, 3], promoted by the MoH in 2004 and 2006 and the increase in human resources allocated in health units, prenatal coverage had little, if any, improvement between 2003 and 2011. However, ethnic issues may be responsible for that, as outlined below.

It is interesting to note that while in 2003 wealth status was associated with prenatal care, in 2011, ethnicity was the major associated factor. The reason for such a change relates to the internal migration process that seems to have happened between 2003 and 2011, when indigenous women left rural and riverine areas and moved to the urban area. Assis Brasil has several indigenous tribes in its territory, much more when comparing to non-indigenous cities in the state of Acre (0.60% of indigenous population in the state and 14.40% of indigenous people in the urban area) [11]. This migration is probably related to new opportunities in health care access, trading and food acquisition in the urban area brought by the construction of the road in 2003. At the same time, non-indigenous women had much more access to prenatal care than indigenous women for the period 2006–2011. There is an exclusive health care system for indigenous people, and the results suggest a lack of efficiency in this particular system. In contrast, the health service attending non-indigenous women is able to incorporate almost all pregnant non-indigenous women. And yet the fact that prenatal care for indigenous women had higher coverage between 2008 and 2010 than 2005 and 2007 suggests that the migratory movement was higher in these later years, or that the indigenous health care system was even more deficient between 2005 and 2007.

The role of ethnicity in prenatal care in the Amazon has already been described by Coimbra et al. (2013) [16] during a national survey about indigenous health. In all regions in the country, indigenous women had less access to prenatal care in recent years than non-indigenous women. The average coverage for indigenous women living in the North region was only 33.4%, considering also women living in tribes and more remote areas. This coverage is much smaller than national average coverage for non-indigenous women, which was reported to be 98.09 % in 2010 [4].

### Number of prenatal care consultations

According to the MoH data, the frequency of women having at least six prenatal consultations in Assis Brasil in 2003 (27.39 %) was lower than the average in the state (33.99 %) and much lower than the national average (51.08 %) for the same year [4]. This means that although many women had access to prenatal care, the number of appointments was low. In 2011, more women had access to at least six prenatal consultations (34.34 %); this coverage was higher than the coverage achieved in Acre (28.78 %), but still lower than the national results (58.50 %) [4], showing that the quality of prenatal care did not change much over the years. It is important to note that the MoH recommendation is different from WHO Millenium goals, which recommends at least four consultations during pregnancy [17]. Maternal education was not associated with attending prenatal care, but it was associated with the total number of consultations during prenatal care. Several other studies have shown association between maternal education and total number of prenatal consultations in Brazil [18–21]. This reflects the importance of education in maternal adherence to prenatal care, probably because education makes it easier for the mother to understand the benefits of attending prenatal consultations.

Having lower socioeconomic status was associated with a smaller number of prenatal consultations. A possible explanation is that in this socioeconomic strata, women have less free time to go to health clinics because of work, or that they cannot afford losing working hours to go to public health posts. Other studies have also identified the association between number of prenatal consultations and socioeconomic status. For instance, Puccini et al. (2003) [22] have shown that salary wages smaller than the minimum national wage were associated with having attended less than six prenatal consultations.

The increase in the number of prenatal consultations according to maternal age in 2011 can be explained by a better understanding of the importance of prenatal care among older women. Puccini et al. (2003) [22] have also found an association between attending less than six consultations and being younger than 20 years old.

On the other hand, a smaller number of consultations among indigenous women in Assis Brasil is possibly the result of temporary migrations, lack of permanent access to health service and cultural issues. An example of a cultural issue hampering prenatal care among indigenous women is that some indigenous women and their spouses do not allow gynecological examination, for instance, even when performed by a woman health worker. Other cultural issues is that some ethnic groups believe western medicine can make them sicker. In a national survey about indigenous health, the percentage of indigenous women with at least six prenatal consultations was 10.9% in the North region and 48.3 % in the Northeastern region, an incredibly low coverage associated with ethnicity [16].

In a study performed in Rio de Janeiro, located in southeast Brazil, Leal et al. (2005) [23] also identified an association between ethnicity and poor prenatal care, where non-Caucasian women (black and Afro-American women) had less access to prenatal care than Caucasian women.

These variables (low maternal education, low wealth status) and others that reflect social inequities (high number of pregnancies and maternal age) are also associated with inadequate prenatal care in other areas of Brazil. Coimbra et al. (2003) [24] have shown this result in the Northeast region, and Bernardes et al. (2014) [25] also found an association among low maternal education, maternal age and parity and prenatal care adherence in the Northeast region. Other studies have shown that parity may influence in adherence to prenatal care consultations, even increasing it, or associated with lower attendance, depending on other cultural aspects of the study population [26]. In some parts of Brazil, ethnicity also plays a role in prenatal access, with indigenous and black women being those more affected by these social and health inequities.

Health literacy, defined as the cognitive and social skills which determine the motivation and ability of individuals to gain access to, understand and use information in ways which promote and maintain good health [27], has been proved to be linked to health behavior [28–30]. In other words, knowing about disease processes may help patients to understand the importance of such healthy behaviors and promote adherence to them. Therefore, health education is needed in all health topics, and in the case of prenatal care, education about the benefits of prenatal care and the benefits of hospital child delivery is constantly needed. How to bring this knowledge in a practical manner to women that have low education in general, such as poor Amazonian women, is a major challenge. Current practices such as posters and announcements in radio and television programs may not reach all women. Health care workers may be the most efficient way of delivering health literacy to these women and promote adhesion to health programs, since they are supposed to visit women and their families frequently during work. Community health workers have been officially employed in Brazil since 2002, to promote health issues among the country, and they have been shown to be able to promote social and cultural changes among patients [31]. However, even health workers must receive proper training in order to promote health literacy among women. Souza et al. (2011) [32] showed that pregnant women using the public health system in Parana, Brazil, complained about the lack of educational actions related to prenatal care while attended by health care workers. In other words, reaching 100% prenatal coverage and institutional child delivery may happen with the aid of such health workers.

### Child delivery

Brazil had an astonishing increase in the number of cesarean surgeries in the past years, with a national average of 40.08 % in 2003, and 24.34 % for the state of Acre [4]. In Assis Brasil, the average was only 17.22 % of childbirths between 1997 and 2003, probably because the city did not have proper facilities or qualified personnel for performing surgery. All cesareans were performed in a neighboring city almost 1150 km away, and until 2003 there was no paved road. The construction of the Pacific road, however, did not result in a large increment in cesarean rates, since the rate was only 20.87 % between 2006 and 2011. This suggests that patients were referred to a neighboring city to receive cesarean surgery only when medically necessary.

The low prevalence of having childbirth with a trained health professional at a hospital facility in Assis Brasil, both in the 2003 and 2011 study, when compared to the national average (96.58 % in 2003 and 97.85 % in 2009), suggests that in-house childbirth managed by midwives or family members continues to be adopted by many pregnant women, either because of cultural aspects or lack of health care access.

The association of year of childbirth with having a child at a hospital facility with trained health professionals in the 2011 study probably reflects the fact that the road construction facilitated fast access to the hospital, both for the patient and health personnel, and at the same time made the city more attractive for health workers to live in. In a qualitative study performed in 2011, inhabitants of Assis Brasil living in the city before and after the construction of the road reported that before the road was paved patient referral to other cities depended upon helicopter transfer or a 3-day journey, making it almost impossible to happen in emergency situations such as birth delivery (“The buses would stay several days in the unpaved road, the pregnant women that were being sent to deliver their babies in other cities did not have time to arrive there, they need a cesarean surgery and would die during the trip because there was not enough time to get to the hospital due to the conditions of the road”) [8]. In addition, maternal education higher than 4 years was also associated with increased odds of giving birth at a hospital, highlighting the importance of education in health issues. Interestingly, while older women tended to attend prenatal care more frequently, they were less likely to seek a hospital for childbirth. One possible explanation is that although they understand the importance of prenatal care and seek it more frequently, there might not be enough time to rush to a hospital due to shorter labor time in some pregnancies.

The difference between evolution of prenatal coverage (93 ~ 95 %) and institutional delivery (17 ~ 20 %) between 2003 and 2011 is high. While pregnant women are easily getting access to prenatal care, most child deliveries are not performed at heath facilities. This can be explained due to the characteristics of the Brazilian health care system and local characteristics. Both doctors and nurses provide prenatal care, so when a medical doctor is not available a nurse provides consultation. The Ministry of Health puts a lot of emphasis in prenatal care coverage and many times only distributes the monthly health stipend for the Municipalities when the health care system reaches certain coverages stipulated by the Government, so this is one of the reasons why coverage was high.

However, child delivery is a different situation. While prenatal care consultations can be performed with a scheduled data, child delivery is at random, and it not always possible to rush the mother to the health care facility. Even when residing in the urban area, the local population moves from urban to rural areas and may be not easy to reach the hospital in time for birth delivery. A second possible explanation is the lack of health professionals to perform birth delivery, especially at night. A third and probably the most important explanation is the local culture of having birth with ‘doulas’ or traditional birth attendants’. This is a long-standing habit of the Brazilian population living in small communities or in the countryside. Most of these women have decades of experience, are long known by the inhabitants and are considered great professionals, even without formal health care education. These explain why they are more trusted than unknown doctors or recently graduated nurses. This phenomenon not only happens in Acre, but is also described in other Amazonian states, such as Tocantins [33], Para [34], Rondonia [35] and Amazonas [36].

### Study limitations

There are a couple limitations in this study. It is possible that a few significant associations were not detected in 2003 due to the small sample size. The second limitation is that it was not possible to identify missed pregnancies via health records, and therefore pregnancies that led to fetal death or stillbirths were not assessed. This bias is probably small because neonatal deaths in the Amazon were estimated at only 1.62 % from 1997–2000 and 0.97 % for 2010 [4]. Another possible source of bias is the inability to track all children that moved out of the urban area during the study period, and thus the results may not portray all associations that happened. Finally, since this is a cross-sectional study, the associations detected do not indicate causality.

## Conclusions

Maternal health indicators for Assis Brasil in 2003 were all below national levels, both regarding prenatal care and childbirth assistance. The comparison between results from the 2003 study and 2011 study shows an increase in health care workers and health units, but the only real improvement in maternal health care was having childbirth at a hospital more frequently. It is possible that the road construction helped to bring more health personnel to the city, especially medical doctors, thus resulting in better childbirth coverage. However, the proportion of women accessing prenatal care did not show marked improvements, as one would expect. The construction of the road probably fostered the migration of indigenous women to the urban area of the city; however, this migration was not properly handled by the health care system. These results show that despite economic and governmental investments, maternal health care in small, geographically isolated towns in the Amazon is still inadequate. Factors associated with this inadequate health care are similar to those previously described in other regions of Brazil: low maternal education, low socioeconomic status and belonging to ethnic minorities. The strengthening of family health programs is still needed in this part of the country.

## Abbreviations

AIC, Akaike’s information criterion; CI, confidence interval; HDI, human development index; MoH, Brazilian Ministry of Health; OR, odds ratio; PCA, principal component analysis
